# Paleoecological and Taphonomic Implications of Insect-Damaged Pleistocene Vertebrate Remains from Rancho La Brea, Southern California

**DOI:** 10.1371/journal.pone.0067119

**Published:** 2013-07-03

**Authors:** Anna R. Holden, John M. Harris, Robert M. Timm

**Affiliations:** 1 Department of Entomology, Natural History Museum of Los Angeles County, Los Angeles, California, United States of America; 2 George C. Page Museum, Los Angeles, California, United States of America; 3 Department of Ecology and Evolutionary Biology and Biodiversity Institute, University of Kansas, Lawrence, Kansas, United States of America; University of Kansas, United States of America

## Abstract

The La Brea Tar Pits, the world’s richest and most important Late Pleistocene fossil locality, offers unsurpassed insights into southern California’s past environments. Recent studies at Rancho La Brea document that insects serve as sensitive and valuable paleoecological and taphonomic indicators. Of the thousands of fossil bird and mammal bones recovered from the Tar Pits, insect trace damage is thus far almost exclusively confined to the foot bones of large herbivores, especially bison, camel, and horse species. Our laboratory experiments with dermestid and tenebrionid beetles establish that the larvae of both consume bone, producing different characteristic feeding traces and providing the first documentation that tenebrionids consume bone. The presence of carcass-exploiting insects in the Rancho La Brea biota provides insight into the taphonomy of the asphaltic bone masses and the environmental conditions under which they accumulated. The succession of dermestids, tenebrionids, and indeterminate traces on many of the foot elements, combined with the climate restrictions and life cycles of these insects, indicate that carcasses could remain unsubmerged for at least 17–20 weeks, thus providing the most reliable estimate to date. Attribution of these traces also suggests that the asphaltic fossils only accumulated during warmer intervals of the Late Pleistocene. Forensic studies need to reevaluate the role of tenebrionids in carcass decomposition and other additional insects that modify bone.

## Introduction

The asphalt seeps of Rancho La Brea are the surface emanations of the Salt Lake Oilfield that trapped countless unwary animals during the course of the past 50,000 years. Natural asphalt can be extremely sticky and we know from other localities that seeps of only a few centimeters in depth suffice to immobilize large domestic animals such as cows and horses. It seems likely that the larger Pleistocene mammals became trapped in the shallow seeps that were at least partly disguised by leaves or windblown dust. Although the vents themselves remain liquid throughout the year, the surface asphalt spreads laterally from the vents and hardens in cooler temperatures. It only remains (or, after hardening, becomes) soft and sticky when the ambient temperature exceeds about 18°C (pers. observ., JMH). This provides a lower temperature constraint for entrapment.

Large mammals trapped in the asphaltic seeps of the Late Pleistocene site of Rancho La Brea were evidently subjected to carnivore ravaging, but few bones show signs of weathering, indicating fairly rapid burial after death and disarticulation [1]. Almost all bones have been separated from adjoining elements. About 2% of the bones display carnivore modification and even fewer display damage by insects. Nevertheless, insect damage can, where present, indicate rates of decay and by their presence insects provide insight on prevailing climatic and environmental conditions. Insect groups associated with carcasses and represented in the Rancho La Brea biota include beetles, flies, termites, true bugs, and ants, wasps, and bees. At least 17 families of beetles are known to be associated with vertebrate carcasses at some time in their life history [2]–[4]. More than half these families are represented as fossils at Rancho La Brea [5]–[6].

The goals of our investigations were A) to identify which bones display insect damage, B) to establish where possible the identity of the insects responsible for the damage, and C) to investigate what this might mean in terms of carcass history and the environmental history of the asphalt seeps.

Our observations are based on fossil bones with insect traces from Rancho La Brea in the collections of the George C. Page Museum, a branch of the Natural History Museum of Los Angeles County. Damage is virtually restricted to spongy and vascular bone and some is indicative of multiple trace-makers. Apart from a subadult condor claw, all insect-damaged bones observed to date are from herbivorous mammals and, with the exception of an immature cervid scapula, all are manus or pes elements.

Termite and ant fossils have been recovered from Rancho La Brea. However, the characteristic star-shaped marks, edge gnawing, and clusters of sub-parallel striations attributed to termites and ants 7–8 have not yet been observed on bones from the asphalt seeps. The capability of dermestid beetles to modify bone is firmly established [2]–[3], [9], and dermestids have been attributed as trace-makers or likely trace-makers in previous literature [10]–[13]. During the course of our experiments, we have observed tenebrionid larvae modifying bone. Both tenebrionids and dermestids were abundant at Rancho La Brea and most of the trace fossils examined during this study can be attributed to one of these families. Attribution of trace-makers enables paleoecological and taphonomic estimations based on the current geographic distribution, climate restrictions, and life cycles of these insects, as well as the succession of their damage.

## Materials and Methods

### Ethics Statement

No specific permission was required for the collection wild-caught tenebrionid beetles used in live insect experiments. *Tenebrio* species were caught from R. Timm’s personal property (near Lawrence, Kansas), and are not an endangered or protected species.

*Eleodes* species used in live experiments are also not endangered or protected. The larvae used were reared from a vibrant museum colony at The Natural History Museum of Los Angeles County. The colony was reared from specimens collected by Brent Karner, the Director of The Insect Zoo. The original colony was obtained in 2010 from 0.75 km east of Highway 19 on Rio Rico Drive, along the Santa Cruz River in Rio Rico, Arizona. This area is public land, and there are no restrictions on collecting *Eleodes*, and no permits are necessary. The specimens were brought back to the Museum with a #526 transport permit from APHIS-PPQ, a division of the U.S. Department of Agriculture (USDA).

All fossil specimens (listed with complete specimen numbers in Appendix S1 and S2) are located at the George C. Page Museum, 5801 Wilshire Boulevard, Los Angeles, California, 90036. No permits were required for the described study, which complied with all relevant regulations.

### Methods

Bones with damage that may have been caused by insects were observed under 40× magnification to eliminate surficial marks from excavation and mechanical preparation and to document those made by larval insects. Mechanical damage included symmetrical puncture marks, adjacent cracks, and areas of bone that were clearly penetrated and/or crushed by preparation tools. Traces attributable to insect damage on fossil bird and mammal bones from Rancho La Brea were compared to damage created by dermestids and tenebrionids on modern wood and bone.

The mandibles of larval *Dermestes maculatus* De Geer, 1774 [14], *Tenebrio molitor* Linnaeus, 1758 [15], and *Eleodes* sp. were imaged and measured. Bones were coated with Spotcheck SKD-S2 Developer and imaged with a macro lens to search for mandibular grooves like those made on modern bone.

### Live Insect Experiments

*Dermestes maculatus* is widely used to clean bones during the preparation of skeletons for osteological study. Many *Dermestes* species are native to California [16; pers. comm. J. Hogue, 5 July 2012] and dermestid fossils have been recorded from Rancho La Brea [6]. We conducted laboratory experiments with *Dermestes maculatus* and comparable experiments using the larvae of the tenebrionid genus *Eleodes*, which today occurs in Los Angeles and southern California and is represented by 17 species or subspecies (at least 550 adult fossils) from Rancho La Brea. Bones were checked for damage at least every three days and the resulting damage was photographed and documented. Comparable feeding trials were conducted at the University of Kansas using both larval and adult *Tenebrio molitor* Linnaeus, 1758 [15] from a commercially purchased colony and wild caught individuals of *Tenebrio obscurus* Fabricius, 1794 [17] from northeastern Kansas.

## Results

Traces attributable to insect damage on fossil bird and mammal bones from Rancho La Brea are comparable to damage created by dermestids and tenebrionids on modern bone in our laboratory colonies. We did not observe scratches on Rancho La Brea bones that could be directly attributed to mandibular grooves, however, these were likely originally present, but subsequently abraded during or following fossilization.

### Dermestid Damage to Bone

To assess dermestid damage on bone, *D*. *maculatus* larvae were fed immature chicken bones and pork ribs. Damage was the result of mandibular “drilling” and occurred on the softer and spongy areas of bone. Mandibular marks were not detected under 40× magnification and we could not distinguish lipid mining from pupal chambers on the basis of morphology. The categories of damage are presented in Table S1.

### Tenebrionid Damage to Bone

Larval *Tenebrio* and *Eleodes* were provided with juvenile chicken bones, pork ribs, juvenile pig metatarsi, and adult horse and sheep sesamoids. Damage is concentrated in more vascular bone with bone mining favored over soft tissue consumption. The ribs and metatarsi received the least modification but were well ossified, more so than any insect-damaged fossil bone. The adult sheep and horse sesamoids were also well ossified and thus, remained untouched except for linear groves and surficial mining.

Mandibular marks from both *Eleodes* and *Tenebrio* are visible under 40× magnification on the bone surface as shallow and linear grooves and also in shallow surficial mining. In contrast to the pitted appearance of dermestid mining, tenebrionids create relatively smooth-surfaced traces, the result of consistent and extensive gradual bone removal. The resulting morphology is categorized in Table S2.

### Attributing Trace Fossil Morphology

Very few insects have been reported to modify bone. Dermestid beetles followed by termites are the groups to which bone damage has most frequently been attributed in the literature [12]–13. Beetles from the family Trogidae also have been reported, but not documented to consume bone [18]. Fejfar and Kaiser [7] stated that tenebrionids modify bones citing the work of MacFarlane [19] and Zacher [20]; however, both authors only note that tenebrionids were attracted to carcasses. Thus, tenebrionids were previously thought to scavenge only soft tissue of carcasses [3]–[4]. In our experiments with both *Eleodes* and *Tenebrio*, we found they also modified bone and often mined it in preference to consuming cartilage, muscle, skin, hair, and tendons. We found that dermestid and tenebrionid larvae usually leave different kinds of damage in different stages of carcass decay.

Our experimental observations document that larvae of both dermestids and tenebrionids leave distinctive traces on the most spongy and vascular areas of bone. Some traces are similar in appearance. For example, both groups expand foramina and both mine areas of cancelleous tissue so that the cylindrical feeding burrows of tenebrionids are almost indistinguishable from dermestid pupal chambers. However, some traces are readily distinguished. Tenebrionid teeth are at least four times larger than those of dermestids, allowing tenebrionids to exploit larger areas of bone at a faster rate, thereby inflicting greater damage. Only tenebrionids produce mandibular marks in the form of linear grooves or surficial mining on bone. Dermestids mine the ends of bone by means of hemispherical pitting and circular bores in contrast with the more even, sideways planing characteristic of tenebrionids. Although, the larvae of both families produce irregular grazing traces along the edges of flat bones, dermestid damage includes scalloping from conjoined hemispherical pits.

### Insect-Modified Fossil Bone

Traces we attribute to dermestids include scalloped quarries and small and medium pits that are often closely grouped together and are of similar size to those created in bone by modern dermestid larvae. Traces we attribute to tenebrionids comprise surficial mining plus extensive and deep quarrying. In many specimens, dermestid pits were subsequently modified by tenebrionid mining. Pits and bores 1.5–2.5 mm in diameter could not confidently be attributed to either dermestid or tenebrionid damage. Some bores and channels are smaller than those made by our experimental dermestid and tenebrionid colonies and may represent different (smaller) trace makers. None of the fossil bones from Rancho La Brea display dermestid or tenebrionid mandibular marks. We believe these were originally present and subsequently removed by abrasion from sediment particles in the surrounding matrix or possibly during preparation. Accordingly, all bone surfaces, including spongy areas, appear polished. A summary of insect damage to bone is presented in Appendix S1 and includes estimates of minimum time taken to produce this damage once large populations of larvae are established. Additional details and measurement of insect traces are presented in Appendix S2.

### Remarks

**Figure 1:**
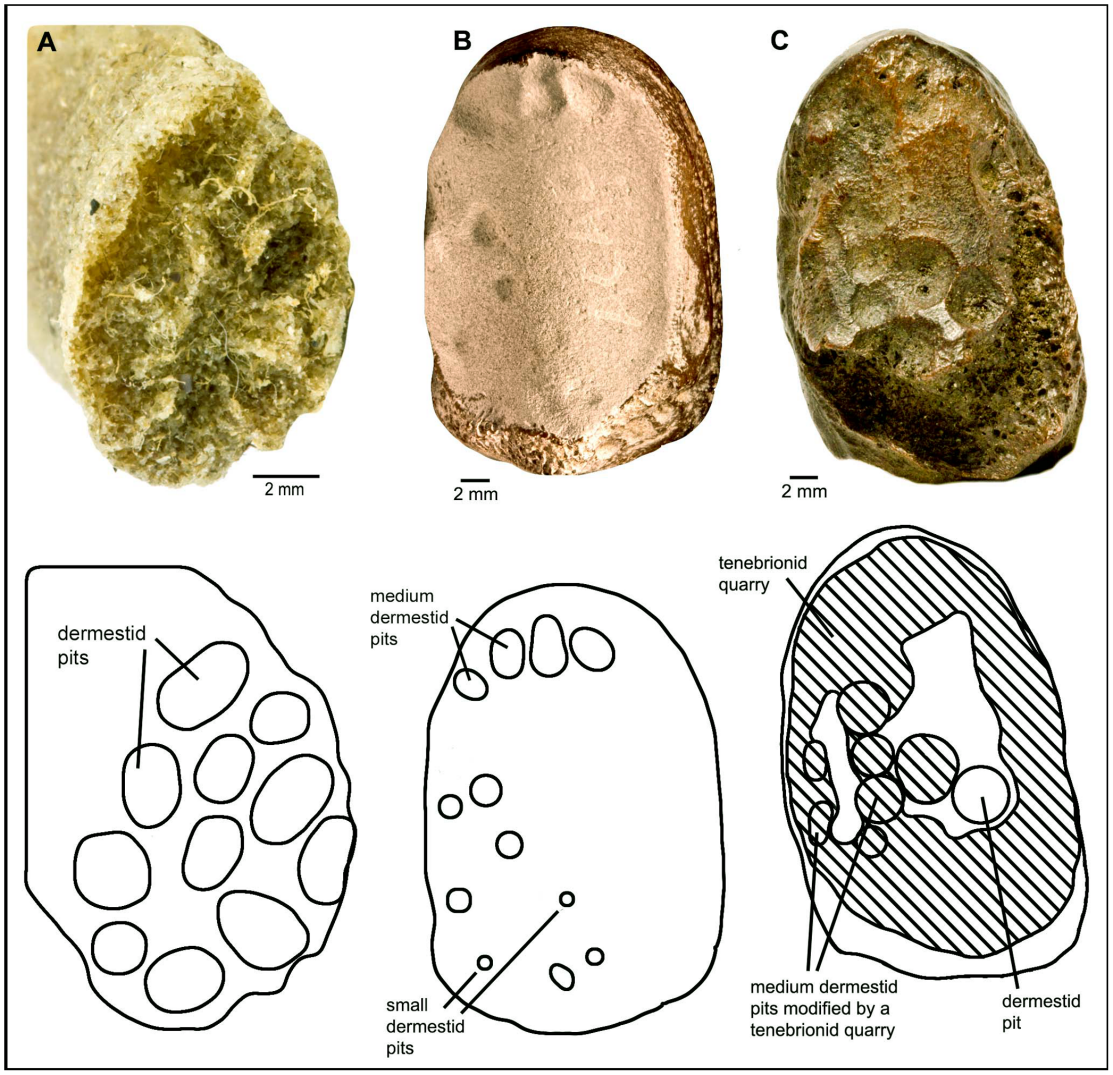
Dermestid damage. **A**: distal juvenile pig rib with medium dermestid pits (experimental bone). **B**: LACMHC 140714, bison left proximal sesamoid coated with Spotcheck to show small and medium dermestid pits (Page collection). **C**: LACMHC 140712, bison left proximal sesamoid with small and medium dermestid pits overlapped by tenebrionid quarry (Page Museum collection).

**Figure 2:**
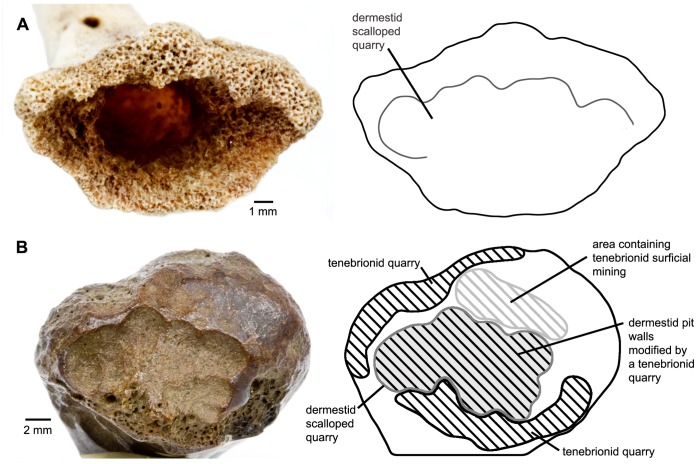
Dermestid damage. **A**: juvenile chicken distal right tarsometatarsus showing bore surrounded by scalloped quarry (experimental bone). **B**: LACMHC Z1835, camel proximal sesamoid displaying dermestid scalloped quarry modified by tenebrionid surficial mining and overlapped by tenebrionid quarry (Page Museum collection).

**Figure 3:**
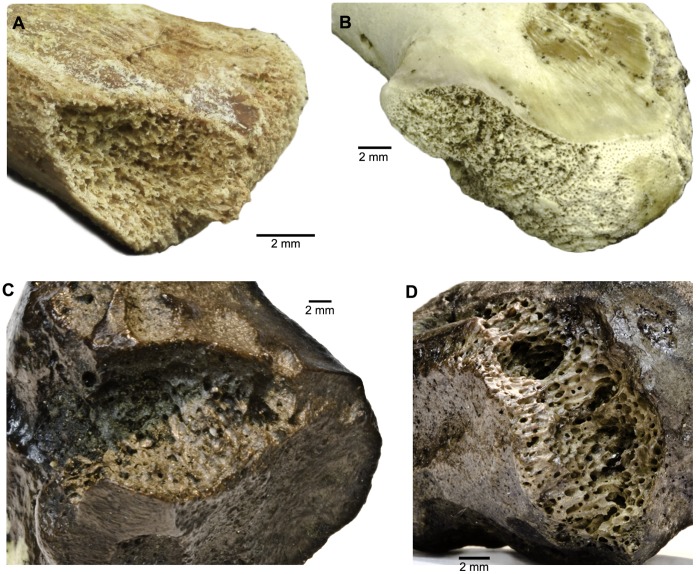
Tenebrionid damage. **A**. juvenile chicken proximal right coracoid with tenebrionid quarrying (experimental bone). **B**: juvenile chicken proximal left coracoid with tenebrionid quarry (experimental bone). **C**: LACMHC 26463, equid proximal right sesamoid with tenebrionid quarry (Page Museum collection). **D**: P23-11272, bison proximal sesamoid showing tenebrionid quarrying (Page Museum collection).

**Figure 4:**
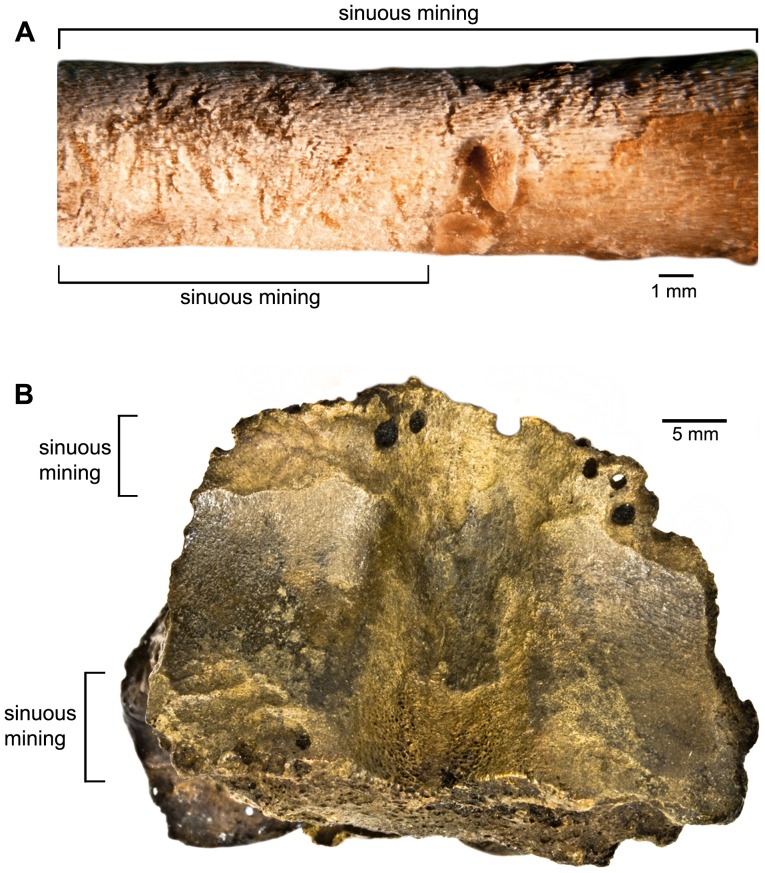
Tenebrionid damage. **A**: juvenile chicken proximal right coracoid with tenebrionid surficial mining on left and tenebrionid bore in the proximal diaphysis on right (experimental bone). **B**: P23-3691, equid proximal first phalanx showing tenebrionid surficial mining (Page Museum collection).

In our laboratory colonies of dermestid and tenebrionid beetles, we were able to reproduce much of the trace morphology observed in the fossil bones. Pits and scalloped quarries made by larval dermestids are consistent in modern and fossil specimens and readily identifiable (Figures 1, 2). Tenebrionid quarries and surficial mining are also similar in modern and fossil examples (Figures 3, 4). Much of the damage observed on the La Brea bones we attribute to tenebrionids, which is consistent with their relative abundance in the Rancho La Brea assemblages and their more flexible environmental requirements.

The Page Museum collections contain more than 1,000 tenebrionids from Rancho La Brea, primarily species of *Eleodes* that outnumber the dermestid remains by a ratio of 10∶1. Although the heavily sclerotized tenebrionid exoskeleton is more likely to preserve in asphalt than the relatively less fortified dermestid exoskeleton, this structural difference alone does not explain the extremely high numbers of tenebrionids, particularly when other well-sclerotized beetles (e.g., ground beetle, weevils, and scarabs) are present but not found in comparable abundance. The preponderance of tenebrionids suggests they were common because they were attracted to, and reproduced on, the decomposing herbivore carcasses entrapped in asphalt.

The genus *Tenebrio* has been reported in forensic studies to show up at carcasses in late and dry stages of decomposition [3] and to eat soft tissue. However, our tenebrionid experiments reveal that, given the opportunity, both *Tenebrio* and *Eleodes* exploit bone at both very early and very late (dry) stages. *T*. *molitor*, generally classified as a stored grain products pest, readily ate bones in our experiments. Given the similarity in feeding behavior of these tenebrionid larvae, it is likely that other genera of tenebrionids also exploit bone.

Britt et al. [12] attributed traces of parallel grooves on Jurassic dinosaur bone to Dermestidae because they have widely spaced mandibles with two apical teeth. However, larval *Tenebrio* also have widely spaced mandibles with two apical teeth. *Dermestes maculatus* has three apical teeth on each mandible, as do larval *Eleodes*. Mandibular marks can be difficult to attribute because tooth structure can be similar between families, yet can vary between genera and species. For example, the ichnospecies *Ostecallis mandibus* [21] includes paired scratching and deeper excavations, some of which are similar to the traces in Britt et al. [12] and Bader et al. [13], but these are difficult to attribute without comparative material from live experiments. Further research on mandible and tooth structure for carcass-exploiting beetles may help attribute trace-makers. Meanwhile, despite the lack of mandibular marks in fossil material, it is clear that tenebrionids are major trace-makers on fossil bone at Rancho La Brea.

Recent significant studies on trace fossils in bone [10], [11], [13], attribute bone damage to dermestids, or suggest that they are the most likely culprit of such modification (these sources also offer references therein of additional attribution to dermestid bone modification). But it is interesting that dermestids are the only beetles reported to consume bone given the vast number of beetle species that exist, many which have well-sclerotized mandibles and share similar carcass-exploiting life-cycles. Whereas dermestids may be responsible for many traces in bone, the use of trace fossils as paleoecological and taphonomic indicators suffers from a lack of sufficiently researched list of possible trace-makers. In many cases, this all-too short list is presented conclusively. More research on potential trace-makers is needed to make accurate identifications and thus, accurate interpretations from trace fossils. It is clearly desirable to use live insect experiments to produce comparative material because mandibular marks can be difficult to attribute; insect mandibular structure can be similar between families, yet can vary between genera and species.

### Other Possible Trace-Makers

**Figure 5:**
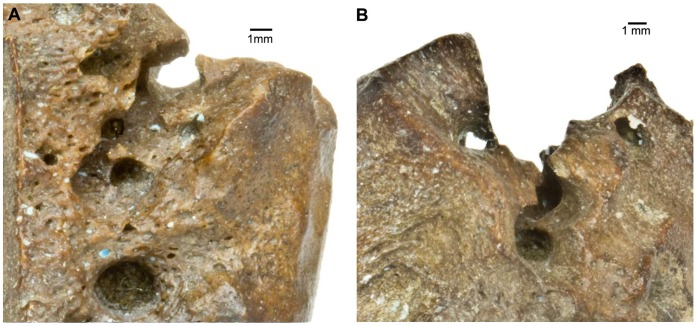
Indeterminate damage. **A**: P23-7744, equid proximal sesamoid showing small circular bores averaging 1.5 mm to 2.5 mm superimposed on a tenebrionid quarry (Page Museum collection). **B**: LACMHC 6335, bison intermediate phalanx, showing indeterminate damage as primary modifications overlapped by a tenebrionid quarry (Page Museum collection).

Many of the insect-damaged La Brea specimens display small, symmetrical circular bores between 1.5–2.5 mm in diameter (Figure 5). These are similar to but smaller than those attributed to dermestids and were not replicated in our dermestid experiments. Although *Dermestes* is the only dermestid known to bore into bone, it is possible that the small bores may have been created by different, smaller dermestids. However, the small size of the bore also suggests the larvae of a small beetle, or possibly a larval fly or moth. This type of damage was superimposed on dermestid and tenebrionid damage to the sesamoids but, interestingly, represented primary damage to the phalanges. Assuming both dermestid and tenebrionid larvae were active during warm to hot intervals, the unidentified trace-maker could represent cooler temperatures in the intervals preceding or following the dermestid and tenebrionid damage.

Given that tenebrionid consumption of bone had not been previously documented, it is conceivable that bone can be modified by other insects with highly-sclerotized mandibles. For example, a species of trogid or hide beetle that has been recovered from the tar pits, *Omorgus suberosus* (Fabricius, 1775) [22], is said to feed on bone [18], [23]. However, this kind of damage has not been documented. Piophilid or cheese flies create pupation chambers in cervid antlers [24]–[25]. They also are one of the most common families associated with carcass decay [26]. Cylindrical holes from the tineid moth genus *Ceratophaga* were recorded in modern bone [27], but this genus has not been recorded from Pleistocene Los Angeles and its current North American distribution is evidently limited to the southeastern United States [28].

## Discussion

Insect species from Rancho La Brea may be viewed in terms of (a) species scavenging on trapped vertebrate carcasses (or preying on the insects exploiting those carcasses [3]–[4], (b) aquatic or semiaquatic insects that were either trapped by asphalt floating on water or alighted in the asphalt mistaking it for water, (c) terrestrial species that inadvertently crawled on the asphalt, and (d) insect fragments from the gut contents and dung of trapped animals [5], 29–31. Insect traces in plants also have been recovered from the asphalt [32; pers. observ., ARH], indicating that insects were brought in by plant material as well. A large proportion of the insect species recovered at Rancho La Brea belong to families known to be attracted to carrion.

Flies are normally the first insects to exploit carcasses whereas beetles follow at a later stage of decomposition. In 1894, Megnin [33] recognized nine stages of corpse decay but in recent years five stages of carcass decay are commonly recognized [3]–[4]. In terms of insects represented at La Brea, blow flies (Calliphoridae) are associated with Stages 1 (fresh carcass) and 2 (bloated carcass). Carrion beetles (Silphidae) appear in Stage 2 to feed on both the carcass and the fly larvae and staphylinid and histerid beetles arrive to feed on the fly larvae. These beetles are joined in Stage 3 (active decay) by dermestid beetle larvae that feed on the carcass; histerids thereafter add dermestid larvae to their diet. In Stage 4 (advanced decay) trogid beetles arrive to feed on dry remains. Stage 5 (dry decay) is characterized by dermestids, clerids, and tenebrionids. Our observations confirm that tenebrionids exploit the carcasses after dermestids. However, our laboratory results clearly indicate that tenebrionid larvae by themselves readily feed on fresh bone.

### Taphonomy

That insect damage was most frequently found on herbivore sesamoids and phalanges is intriguing. Both dermestid and tenebrionid larvae are negatively phototrophic. Dermestid larvae are reported to require tissue with fat near the surface or spliced between muscle bundles and avoid meat that is too lean, dry, or fatty [34] and are known to shelter under mummified skin where they burrow into tendons and bore into joints [35]. The subcutaneous environments adjacent to sesamoids and phalanges meet the pupation requirements for both dermestids and tenebrionids and foramina can be readily enlarged during bone feeding and excavation of pupal chambers. That insect damage was not observed on carnivore phalanges or sesamoids may reflect the relative thinness of carnivore skin (retaining moisture less well and providing less protection and less insulation from the external environment) or could be due to the relatively small size of their sesamoids.

Only a small proportion of camel (5%), bison (3.2%), and equid (1.7%) sesamoids show insect damage, but the distal limbs of which these sesamoids were part must have remained unsubmerged in asphalt for an appreciable period of time. Spencer et al. [1] postulated that larger mammals trapped in asphalt fell over onto their side, allowing predators to ravage and remove the exposed limbs. Presumably the insect-damaged feet were either (1) portions of exposed limbs that were ignored or discarded by scavengers, or (2) trapped in the asphalt but exploited by insects via the unsubmerged proximal portion of the limb. A plausible scenario is that carnivore feeding opened up the thick skin of the herbivore prey for insect access and the distal limb–drying, exposed, and containing little meat–received less attention from vertebrate scavengers than other portions of the carcass. However, the drying skin of the distal limbs trapped moisture subcutaneously, creating a favorable feeding, egg-laying, and pupation microenvironment for scavenging insects that included large populations of tenebrionids and dermestids. The consequent damage to bone, especially to the small sesamoid bones, was a byproduct of their feeding, pupation, and reproductive behavior.

The recognition of traces of three or more insects on some of the examined bones points to sequential exploitation of the carcass, raising the question of how long the carcasses remained available before being submerged by the asphalt or before cooler conditions terminated insect activity. Stock [36] estimated that the diversity of carcass-exploiting insects from Rancho La Brea indicated the carcasses remained available for at least 5 months. Insect-damaged bones provide an opportunity to refine this estimate.

The best estimation of the duration of the annual warm season is indicated by specimens with the most significant and varied damage. For example, an *E. occidentalis* intermediate phalanx **(**LACMHC Z3885) displays tenebrionid damage estimated at 9–12 weeks plus five dermestid bores, and damage from indeterminate insects. If made by tenebrionids, the five dermestid bores would have taken 3 weeks to produce. However, dermestid damage occurs at a comparatively slower rate than tenebrionids, at least partially due to their smaller mandibles, so the dermestid damage is estimated at closer to 5 weeks. To this interval of 14–17 weeks must be added the time taken for dermestid eggs to develop and hatch (2–10 days [9]) or *Eleodes* eggs to develop and hatch (a minimum of 6 days and average of 14.5 days [37]), and for both to flourish into large populations. No firm time estimate can be made for the indeterminate insect damage, but it is unlikely that any insect could create such damage in a matter of a few days and an interval of several weeks is more likely. Thus, this specimen sustained damage that represents a passage of at least 17–20 weeks. That dermestids first appear during the active stages of decay and tenebrionids appear during the dry stages of decay extends the minimum time estimate further as time would have elapsed while the ambient temperature warmed up prior to the appearance of the dermestids.

### Paleoecological Inferences

Predaceous and scavenging beetles such as dermestids and tenebrionids are valuable for paleoenvironmental reconstructions because these insects are not restricted to specific vegetation and are able to readily migrate to appropriately warm or cool regions when climatic change occurs [38]–[40]. It may take more than a few hundred years for temperature changes to be captured in palynological records [39], whereas the mobility of insects can reflect climate change occurring in a matter of decades [40]–[41].

Dermestid larvae require moist flesh and high ambient temperature and humidity. Although cosmopolitan in distribution, *Dermestes* species are most active during the warmer months [4]. Experiments suggest that dermestids require 60–80% humidity, the larvae do not pupate to adulthood below 25°C and that the optimum temperature for growth is 30°C [42]. In our laboratory colonies, larval *Eleodes* were less environmentally sensitive and were maintained outdoors where temperatures fluctuated between 12°C and 30°C daily in Los Angeles (21–29°C for both *Tenebrio molitor* and *T. obscurus* in Kansas). They consumed bones with equal vigor whether these were provided immediately after meat was removed or after a month of drying. The more than 100 extant *Eleodes* species typically occur in semiarid and desert areas of the western United States [43] where they are exposed to high temperatures and low humidity [44].

If the overall temperature in southern California was distinctly cooler than today [45], the asphalt would only have been an effective trap for a relatively short ( = warm) part of the year. Unsubmerged skeletal elements could be exposed for two or more years, in which case at least some weathering would be expected. However, Spencer et al. [1] indicate the large mammal bones from Pit 91 were buried fairly rapidly with 93% of the bones showing no weathering or only some cracking and exfoliation. Many beetle species trapped at Rancho La Brea during the late Pleistocene are today distributed in the warmer areas of North America (and Mexico) and some insect damage to the fossil bones is consistent with an annual warm interval of at least 4–5 months at the time that the fossils were trapped.

The temperature requirements of dermestids and tenebrionids conflict with interpretations of Late Pleistocene climatic conditions in southern California based on pollen from deep sea cores. Heusser [44] concluded that during Oxygen Isotope Stage (OIS) 3 (59–24 ka) the mean annual temperature in southern California was in the order of ∼11°C but decreased during the Last Glacial Interval (LGI) (24–14 ka) to ∼5°C. Precipitation estimates similarly decrease from ∼100 cm in OIS 3 to ∼45 cm in the LGI. It is interesting, therefore, that the majority of insect species from Rancho La Brea are today found in the warmer parts of the United States and Mexico.

The current geographic distribution and climate restrictions of insect species preserved at Rancho La Brea, together with the physical properties of natural asphalt, indicate that the fossil insects represent warmer intervals of the Late Pleistocene. Short term increases in temperature that would result both in increased asphalt entrapment and in the short-lived invasion of warm temperature insects over intervals of decades to a few hundred years would not necessarily be captured in the offshore palynological record.

Thus, the peak intervals of entrapment are likely to have occurred primarily during warmer intervals when the seeps were more active, trapping temperate to warm-adapted insect and vertebrate species, and resulting in carcasses that could be exploited for more than a few months per year. The apparent clustering of dated specimens [46] suggests some periodicity of warmer interludes during the last glacial interval.

## Supporting Information

Table S1 Sequential stages of dermestid bone damage in live experiments.
(DOCX)

Table S2 Sequential stages of tenebrionid bone damage in live experiments.
(DOCX)

Appendix S1 **Summarized description of insect damage and estimates of minimum time to produce damage.**
(DOCX)

Appendix S2 **Detailed description of insect damage.**
(DOCX)
