# Supplementary material for: Paleoecological and Taphonomic Implications of Insect-Damaged Pleistocene Vertebrate Remains from Rancho La Brea, Southern California
Source: PLoS One. 2013 Jul 3;8(7):e67119. doi: 10.1371/journal.pone.0067119 (PMC3700975; doi:10.1371/journal.pone.0067119)
Supplement: Table S1 — Sequential stages of dermestid bone damage in live experiments. (DOCX) [file pone.0067119.s001.docx]

| **Term** | **Definition** |
| --- | --- |
| Small Pit | Circular, with concave bases, 1.5–2.5 mm in diameter and 0.75–2.5 mm deep (Figure 1B). |
| Medium Pit | Circular, with concave bases, about 1.5–2.5 mm in diameter and 2.5–5.5 mm deep. Scalloped contours of bone removal created by conjoined pitting (Figure 1B). |
| Bore | Tunnels between 2.5–3.5 mm in diameter, over 5.5 mm deep, sometimes conjoined and extending to the width of the bone and removing all cancellous bone (Figure 2A). |
| Scalloped Quarry stage 1 | Edge grazing on innominate up to 1 mm deep; scalloping due to conjunction of hemispherical pits. |
| Scalloped Quarry Stage 2 | Edge grazing on innominate between 1–2 mm deep. |
| Scalloped Quarry Stage 3 | Edge grazing on innominate over 2 mm deep (Figure 2A). In all quarry stages, mining penetrated more deeply into the spongy interior of bone and often included visible pitting. |
