# Supplementary material for: Paleoecological and Taphonomic Implications of Insect-Damaged Pleistocene Vertebrate Remains from Rancho La Brea, Southern California
Source: PLoS One. 2013 Jul 3;8(7):e67119. doi: 10.1371/journal.pone.0067119 (PMC3700975; doi:10.1371/journal.pone.0067119)
Supplement: Table S2 — Sequential stages of tenebrionid bone damage in live experiments. (DOCX) [file pone.0067119.s002.docx]

| **Term** | **Definition** |
| --- | --- |
| Linear Groove | Shallow, asymmetrical and linear mandibular marks about 0.2 mm wide, often overlapping in various directions and most often located on the epiphyses. |
| Bore | Enlarged foramina creating tunnels extending more than 6 mm, entering into subsurface spongy bone and often completely removing it. |
| Quarry Stage 1 | Smooth, surficial patches 1–2 mm in diameter located in epiphyses. |
| Quarry Stage 2 | Smooth surficial patches from 2–5 mm in diameter located on edges and epiphyses. Arcs of bone up to 5 mm wide and 4 mm deep removed from thin and/or flat bones. |
| Quarry Stage 3 | Epiphyses almost entirely removed and mining continues onto the diaphysis. |
| Surficial Mine | Overlapping mandibular scratches remove a thin layer of surface bone over large areas that then have a consistent, shredded texture (Figure 4A). |
