# Supplementary material for: Paleoecological and Taphonomic Implications of Insect-Damaged Pleistocene Vertebrate Remains from Rancho La Brea, Southern California
Source: PLoS One. 2013 Jul 3;8(7):e67119. doi: 10.1371/journal.pone.0067119 (PMC3700975; doi:10.1371/journal.pone.0067119)
Supplement: Appendix S1 — Summarized description of insect damage and estimates of minimum time to produce damage. (DOCX) [file pone.0067119.s003.docx]

APPENDIX 1. Summarized description of insect damage and estimates of minimum time to produce damage. All specimens are reposited at the George C. Page Museum, 5801 Wilshire Boulevard, Los Angeles, California, 90036.

**LACMHC 6335, *Bison antiquus*, Left Intermediate Phalanx, Pit 61**—Tenebrionid surficial mines occur over 19% of the anterior and posterior surfaces with mining extending deeply and extensively into the cancellous tissue (10–12 weeks). No dermestid damage is evident but would have been modified by tenebrionid quarries. Many small bores from an unidentified insect occur on all surfaces except distal. Bone mass removed: 50%.

**LACMHC 6336, *B. antiquus*, Right Intermediate Phalanx, Pit 61—**A dermestid bore is present on the posterior surface. Tenebrionid quarrying removed 13% of the anterior surface and extends into the cancellous tissue (3–5 weeks). Other significant damage includes numerous bores, averaging 1–2 mm in diameter, on all surfaces by an unidentified insect. Bone mass removed: 15%.

**LACMHC 6338, *B. antiquus*, Right Intermediate Phalanx, Pit 67—**Isolated dermestid bores occur on all but the posterior surface. Tenebrionid quarrying removed 23% of the bone, mostly from the anterior surface, and extends into the center of the cancellous tissue (6–8 weeks). Two small bores from an unidentified insect occur on the medial side. Bone mass removed: 30%.

**LACMHC 140711, *B. antiquus*, Right Proximal Sesamoid (?), Pit 3—**No dermestid damage but 61% of surface area shows tenebrionid quarrying (2–3 weeks). Bone mass removed: 9%.

**LACMHC 140712, *B. antiquus*, Left Proximal Sesamoid (Manus), Pit 3**—4% of surface area shows dermestid damage in the form of closely spaced medium pits (Figure 1C). Tenebrionid quarrying occupies 73% of the surface area (3 weeks). Bone mass removed: 16%.

**LACMHC 140713, *B. antiquus*, Left Proximal Sesamoid (Pes) Pit 1**—Three conjoined dermestid pits occupy 5% of surface area. Tenebrionid quarrying occurs over 47% of the surface area (2 weeks). Bone mass removed: <2%.

**LACMHC 140714, *B. antiquus*, Left Proximal Sesamoid, Academy Pit—**Dermestid damage in the form of eleven small pits, some closely spaced, occupies 6% of the surface area (Figure 1B). Tenebrionid mining and surficial quarries occur over 26% of the surface area (1 week). Bone mass removed: <2%.

**LACMHC 26463, *Equus occidentalis*, Proximal Sesamoid, Pit 67—**Tenebrionid quarrying extends over about 44% of the surface area (2–3 weeks) (Figure 3D). Bone mass removed: <5%.

**LACMHC B7986, *Gymnogyps* sp., Digit Three Phalanx Two (pes), Pit 61—**A single bore could represent either dermestid or tenebrionid damage.

**LACMHC R50179, *E. occidentalis*, Proximal Sesamoid, Pit 91**—Three partial dermestid bores on the anterior and lateral surface are overlapped by tenebrionid quarries that occupy 60% of the surface area, mostly on the anterior surface (5–7 weeks). Four small bores from an undetermined insect, between 1.58–2.5 mm in diameter, occur on the anterior and lateral surfaces. Bone mass removed: 40%.

**LACMHC Z1835, *Camelops hesternus,* Sesamoid (Manus), Pit 3**—Dermestid damage in the form of conjoined medium pits, some with scalloped outlines, occupies 18% of the surface area (Figure 2B). Tenebrionid damage comprising quarries and surficial mining occurs over 58% of the surface area (1–2 weeks). Bone mass removed: <2%.

**LACMHC Z2219, *C. hesternus*, Intermediate Phalanx, Pit 13—**One bore is attributed to dermestids. Three additional bore-like holes could have been created by dermestids or are expanded foramina mined by tenebrionids. Tenebrionid surficial mining and quarries occur on all surfaces and total 67% of surface bone removal (6–8 weeks). Bone mass removed: 25%.

**LACMHC Z3885, *E. occidentalis*, (Left?) Intermediate Phalanx, Pit 3—**Five dermestid bores on the anterior surface are overlapped by tenebrionid quarries extending into cancellous tissue. Tenebrionid quarries occupy 32% of the posterior surface and extend into the proximal, lateral, and medial edges (9–12 weeks). Channels and bores from an unidentified insect between 1.2 mm and 2.3 mm wide occur on the lateral and anterior margin. Bone mass removed: 30%.

**LACMHC Z3932, *E. occidentalis*, Right Intermediate Phalanx, Pit 3—**Two deep dermestid bores at the ventral edge of the proximal epiphysis are overlapped by tenebrionid quarries that occupy the entire medial surface and the posterior/proximal margin (3 weeks). Many small bores and channels by an unidentified insect occur on all surfaces and especially along the proximal epiphyseal edges. Bone mass removed: 8%.

**LACMRLP R31739, *E. occidentalis*, Left Intermediate Phalanx, Pit 91**—Three dermestid bores occur on the posterior edge of the proximal epiphysis. Tenebrionid damage is represented by conjoined patches of sinuous mining on the distal surface (3 weeks). Small bores created by an unidentified insect occur extensively on all surfaces except distal. Bone mass removed: 7%.

**P23-3691, *E. occidentalis*, Right Hind Proximal Phalanx, Box 1—**Dermestid channeling and pits occupy 0.5% of the surface area. Tenebrionid damage in the form of surficial mining and a quarrying occur over 51% of the surface area (2–3 weeks) (Figure 4B). Small channels suggest damage from third (as yet undetermined) insect group. Bone mass removed: <2%.

**P23-5525, *Odocoileus* sp., Juvenile, Proximal Left Scapula, Box 1—**Four bores on the lateral surface cannot be assigned to either dermestid or tenebrionid damage.

**P23-7743, *E. occidentalis*, Proximal Sesamoid, Box 1—**Dermestid damage takes the form of three bores occupying 12% of the surface area. Tenebrionid quarrying occupies 63% of the surface area (6–9 weeks). Additional small bores suggest the presence of a third group of insects. Bone mass removed: ~50%.

**P23-7744, *E. occidentalis*, Proximal Sesamoid, Box 1—**No dermestid damage but 62% of surface area shows tenebrionid quarrying (4–6 weeks) and additional small bores suggest the presence of a third insect group (Figure 5). Bone mass removed: ~33%.

**P23-11272, *B. antiquus*, Left Proximal Sesamoid (Manus), Box 1—**Shallow dermestid pitting on the articular surface occupies 2% of the surface area. Tenebrionid quarrying occurs over 44% of the surface area (2–3 weeks) (Figure 3D). Small bores indicate the work of additional (as yet undetermined) small insect species. Bone mass removed: ~10%.
