# Supplementary material for: Paleoecological and Taphonomic Implications of Insect-Damaged Pleistocene Vertebrate Remains from Rancho La Brea, Southern California
Source: PLoS One. 2013 Jul 3;8(7):e67119. doi: 10.1371/journal.pone.0067119 (PMC3700975; doi:10.1371/journal.pone.0067119)
Supplement: Appendix S2 — Detailed description of insect damage. (DOCX) [file pone.0067119.s004.docx]

APPENDIX 2. Detailed description of insect damage. All specimens are reposited at the George C. Page Museum, 5801 Wilshire Boulevard, Los Angeles, California, 90036.

**LACMHC 6335, *Bison antiquus*, Left Intermediate Phalanx, Pit 61 (Figure 5B)**

**Tenebrionid Damage—**A quarry on the anterior surface, between about 10.0–22.0 mm wide and about 32.0 mm long, enters into the almost entirely mined cavity of cancellous tissue and punctures through to areas on all other surfaces while also extending past the distal and medial edges; a quarry on the posterior surface between 4.0–10.0 mm wide, about 17.0 mm long, overlaps small bores and enters into the almost entirely mined cavity of cancellous tissue, punctures through to areas on all other surfaces; a quarry on the posterior surface about 4.6–7.0 mm wide, 9.26 mm long, overlaps small bores and enters into the almost entirely mined cavity of cancellous tissue, punctures through to areas on all other surfaces.

**Indeterminate Damage—**Small bores on all surfaces except distal, often conjoined and averaging 1.0–2.5 mm in diameter, averaging between 0.9 mm deep, or entering mined cancellous tissue. One channel on the medial surface about 1.2 mm wide, 4.49 mm long, and 1.0 mm deep; one channel on the posterior surface about 2.5 mm wide, 6.48 mm long, and about 1.5 mm deep.

**LACMHC 6336, *B. antiquus*, Right Intermediate Phalanx, Pit 61**

**Dermestid Damage—**A bore on the posterior surface is about 4.2 mm in diameter and 10.44 mm deep; another bore on the proximal surface about 3.9 mm in diameter and about 8.7 mm deep.

**Tenebrionid Damage—**A quarry on the anterior surface between about 5.0–20.0 mm wide, about 30.0 mm long, and between 5.77–12.3 mm deep; a quarry on the posterior surface about 8.3 mm wide, 8.5 mm long, and about 1.0 mm deep, overlaps small bores. Surficial mining on the medial surface about 7.6 mm in diameter and about 0.6 mm deep.

**Indeterminate Damage—**Many small bores on all surfaces averaging 1.0–2.0 mm in diameter, most averaging 1.5 mm deep, some enter into mined cavities of cancellous tissue.

**LACMHC 6338, *B. antiquus*, Right Intermediate Phalanx, Pit 67**

**Dermestid Damage—**A bore on the proximal surface is about 3.4 mm in diameter and 9.4 mm deep; a partial bore on the proximal surface, about 4.6 mm in diameter, conjoined with adjacent bores and enters the almost entirely mined cavity of cancellous tissue; a bore, or conjoined bores on the proximal surface approximately 6.2 mm in diameter, also conjoined with adjacent bores enters the almost entirely mined cavity of cancellous tissue; a bore on the anterior surface approximately 3.0 mm in diameter enters the almost entirely mined cavity of cancellous tissue; a bore on the anterior surface about 3.7 mm in diameter enters the almost entirely mined cavity of cancellous tissue; an elliptical bore on the lateral surface about 3.12 mm wide and 4.6 mm long enters the almost entirely mined cavity of cancellous tissue at an angle. A medium pit on the lateral surface about 3.0 mm in diameter and about 2.5 mm deep.

**Tenebrionid Damage—**A quarry on the anterior surface about 14.0 mm wide and about 28.0 mm long, enters the almost entirely mined cavity of cancellous bone is overlapped by an adjacent quarry; a quarry about 11.5 mm in diameter, 0.25–1.5 mm deep overlaps a bore on the posterior surface; a quarry on the distal surface about 3.4–14.0 mm wide, about 22.0 mm long, and 0.25–1.0 mm deep, is overlapped by an adjacent quarry.

**Indeterminate Damage—**A small bore on the lateral surface, about 1.6 mm in diameter and 3.5 mm long; another bore on the lateral surface, about 1.5 mm in diameter and 3.17 mm deep.

**LACMHC 140711, *B. antiquus*, Right Proximal Sesamoid, Pit 3**

**Dermestid Damage—**None.

**Tenebrionid** **Damage—**A quarry on the posterior surface between 4.47–13.81 mm wide, about 33.0 mm long, and between 0.25–3.65 mm deep abuts the medial and dorsal sides; a quarry on the posterior surface between 3.26–7.69 mm wide, 8.26 mm long, and between 0.25–1.85 mm deep; a quarry on the posterior surface between 6.1–12.62 mm wide, 36.0 mm long, and between 0.25–4.02 mm deep; a quarry on the proximal surface between 5.86–10.08 mm wide is overlapped by an adjacent quarry, about 12.85 mm long, and between 0.25–2.07 mm deep; a quarry on the posterior surface 2.29 mm wide, 5.59 mm long, and about 0.6 mm deep; a quarry on the posterior surface between 2.65 mm wide and 9.74 mm long and about 1 mm deep is overlapped by another, deeper quarry; a quarry on the distal surface about 2.5 mm wide, 12.32 mm long and about 0.6 mm deep.

**LACMHC 140712, *B. antiquus*, Left Proximal Sesamoid (Manus), Pit 3 (Figure 1C)**

**Dermestid Damage**—Five medium pits on the articular surface between about 3.5–5.0 mm wide, up to 1.5 mm deep, very closely grouped, sometimes conjoined.

**Tenebrionid Damage**—A quarry on the posterior surface between 7.2–16.02 mm wide, about 21.0 mm long and between 0.25–5.0 mm deep, although mostly about 2.5 mm deep (depths of 5.0 mm could be the result of an expanded foramen or mining by another species); a quarry between 1.75–9.43 mm wide, and about 56.0 mm long, between 0.25–6.45 mm deep wraps around the medial, dorsal and part of the lateral surface; a quarry on the lateral side between 4.03–10.2 mm wide, about 31.0 mm long, and between 0.25–4.58 mm deep abuts two other quarries; a quarry on the anterior surface between 3.0–7.59 mm wide, about 22.0 mm long, and between 0.25–1.0 mm deep conjoins with dermestid pits.

**LACMHC 140713, *B. antiquus*, Left Proximal Sesamoid (Pes), Pit 1**

**Dermestid Damage—**Three conjoined, medium pits on posterior surface 3.5–4.5 mm wide and between about 0.7–1.73 mm deep.

**Tenebrionid Damage—**A quarry on the medial surface between 3.64–11.10 mm wide, about 22.0 mm long, and between 0.25–0.91 mm deep; a quarry on the dorsal surface between 4.59–10.68 mm wide, about 24.0 mm long, and between 0.25–2.7 mm deep abuts two other quarries; a quarry on mostly the dorsal and a small portion of the medial surface between 1.48–9.03 mm wide, about 24.0 mm long, and between 0.25–1.33 mm deep; a quarry on mostly the posterior surface but also on a portion of the medial surface between 1.51–8.73 mm wide, about 19.0 mm long, and between 0.25–2.22 mm deep; a quarry on the posterior surface impacting three conjoined dermestid pits is between 3.95–4.78 mm wide, 14.41 mm long, and between about 0.7–1.73 mm deep; a quarry on the dorsal surface is between 1.7–4.55 mm wide, 10.11 mm long, and about 0.25 mm deep.

**LACMHC 140714, *B. antiquus*, Left Proximal Sesamoid, Academy Pit (Figure 1B)**

**Dermestid Damage—**Eleven small pits on the articular surface, between about 2.0–2.5 mm wide and 0.5–0.75 deep, some closely grouped together.

**Tenebrionid Damage—**A quarry on the dorsal surface between 1.43–9.3 mm wide, about 23.5 mm long, and between 0.25–1.3 mm deep. A patch of surficial mining on the posterior surface about 7.0 mm wide and 16.0 mm long; another patch of surficial mining also on the posterior surface is about 7.06 mm wide and 7.8 mm long.

**LACMHC 26463,** ***Equus occidentalis*, Proximal Sesamoid, Pit 67 (Figure 3C)**

**Dermestid Damage—**None.

**Tenebrionid Damage—**A quarry on the posterior surface between 2.3–17.0 mm wide, about 24.0 mm long, and between 0.25–2.6 mm deep; a quarry along the edge between the medial and anterior surfaces and between the distal anterior and posterior edge is between 2.6–8.9 mm wide, about 30.0 mm long, and between 0.25–6.5 mm deep; a quarry on the distal surface is between 5.82–9.12 mm wide, about 9.75 mm long, and between about 0.25–0.75 mm deep; a quarry on the anterior surface is 4.6 mm wide, 6.45 mm long, and between 0.25–1.5 mm deep; a quarry between 2.69–16.0 mm wide, about 25.0 mm long, between 0.25–1.5 mm deep covers almost the entire anterior surface and is overlapped by an adjacent quarry.

**Indeterminate Damage—**None.

**LACMHC B7986, *Gymnogyps* sp., Digit Three Phalanx Two (Pes), Pit 61**

**Indeterminate Damage—**A bore 2.95 mm in diameter along the suture line on the ventral surface of the proximal end near the epiphysis that opens onto cancellous tissue. Both dermestid and tenebrionid larvae were observed to create indistinguishable circular bores that enter into similar cavities.

**LACMHC Z1835, *Camelops hesternus*, Sesamoid (Manus), Pit 3 (Figure 2B)**

**Dermestid Damage—**Conjoined medium pits on the proximal, posterior side are 3.5–4.4 mm wide and up to about 2.5 mm deep; these appear to have been later modified by tenebrionids. Some of the pits are visibly circular and present a clear scalloped outline when conjoined shape but subsequent surface removal has eliminated other structural detail.

**Tenebrionid Damage—**Includes a quarry on the anterior surface and lateral edge between 2.34–5.0 mm wide, about 10.0 mm long, and between 0.25–1.52 mm deep; conjoined small quarries on the anterior surface between 1.56–4.5 mm wide, about 12.0 mm long, and between 0.25–0.82 mm deep; a quarry on the proximal, posterior surface between 2.28–5.45 mm wide, about 20.6 mm long, and between 0.25–1.73 mm deep; a triangular patch of surficial mining about 17.67 mm by 17.53 mm by 19.91 mm on the proximal surface overlapping three quarries about 4.25 mm in diameter and ranging from 0.4 mm to about 1.5 mm deep.

**LACMHC Z2219, *C. hesternus*, Intermediate Phalanx, Pit 13**

**Dermestid Damage—**An elliptical bore at an angle on the lateral surface is 3.77 mm by 2.87 mm and 23.0 mm deep entering into the cancellous tissue.

**Tenebrionid Damage—**A quarry about 15.5 mm wide and about 31.0 mm long, and 0.25–1.0 mm deep on virtually the entire proximal surface and overlapped by a quarry on the medial surface; a quarry on the medial surface about 13.0 mm in diameter and at least 18.0 mm deep into the cancellous tissue; a quarry between the proximal and proximal/medial edge between 3.2 mm and at least 13.0 mm wide, about 20.0 mm long, and between 0.25–6.0 mm deep on the edge; a quarry from the medial surface about 12.0 mm in diameter and about 33.0 mm deep into the cancellous tissue; an elliptical quarry on the lateral surface about 10.5 mm wide and 13.4 mm long, extends about 35.0 mm deep into the cancellous tissue; a quarry on the anterior surface is about 6.0 mm in diameter and about 3.7 mm deep. Surficial mining on the posterior surface between about 7.0 and 14.0 mm wide, and at least 20.0 mm long is overlapped by two tenebrionid quarries; surficial mining on the anterior surface about 18.0 mm wide and 28.0 mm long is overlapped by tenebrionid quarries.

**Indeterminate Damage—**Three possibly conjoined bores on the distal surface about 3.5 mm in diameter, or bone eroded by abrasion or weathering to reveal adjacent foramina. Condyles and distal surface are either quarried by tenebrionids and/or eroded by weathering/abrasion.

**LACMHC Z3885, *E. occidentalis*, Left Intermediate Phalanx, Pit 3**

**Dermestid Damage—**Two elliptical bores at an angle on the anterior surface about 4.5 mm wide, 6.0 mm long, and about 7.0 mm deep; a bore on the anterior surface about 5.3 mm in diameter, conjoined by adjacent bores, of no depth because all material except the surface layer is removed; a bore on the anterior surface about 3.8 mm in diameter, conjoined by adjacent bores, of no depth because all material except the surface layer is removed; a bore on the anterior surface about 5.0 mm in diameter conjoined by adjacent bores, of no depth because all material except the surface layer is removed.

**Tenebrionid Damage—**A quarry encompassing most of the posterior surface and along the proximal, lateral, and medial edges from 4.5 mm to about 28.0 mm wide, about 70.0 mm long, and between 0.25–23.0 mm deep (punctures through the anterior surface); a quarry on the anterior surface and along the distal edge is between about 2.4–10.0 mm wide, 63.0 mm long, and between 0.25–1.5 mm deep.

**Indeterminate Damage—**A bore on the medial side about 2.0 mm in diameter and about 3.0 mm deep within tenebrionid quarry or exposed foramina; a bore on the medial/anterior edge continues into a channel about 2.5 mm in diameter, 12.23 mm long; a bore on the medial/anterior edge about 1.2 mm in diameter continues into a channel about 7.9 mm long; a bore on the anterior/medial edge is about 1.2 mm in diameter and about 1.0 mm deep; a bore on the anterior surface is about 2.3 mm in diameter and 5.75 mm deep. A channel on the anterior surface is about 2.0 mm wide, 5.17 mm long, and about 0.9 mm deep; a channel on the medial surface about 1.3 mm wide, at least 6.62 mm long, and about 1.3 mm deep is overlapped by a tenebrionid quarry; a channel on the medial surface about 2.32 mm wide, 6.4 mm long, and about 1.0 mm deep is overlapped by a tenebrionid quarry.

**LACMHC Z3932, *E. occidentalis*, Right Intermediate Phalanx, Pit 3**

**Dermestid Damage—**A bore on the posterior/proximal edge about 4.6 mm in diameter is overlapped by a tenebrionid quarry over 60.0 mm long extending to the anterior surface and curving along the medial/anterior edge; a bore on the medial surface about 4.3 mm in diameter and 10.0 mm long is overlapped by tenebrionid quarries on both ends.

**Tenebrionid Damage—**A quarry encompassing almost the entire medial surface, as well as, part of the anterior surface is between 4.13–20.0 mm wide, about 33.0 mm long, and between 0.5–4.5 mm deep; a quarry on the posterior/proximal edge about 9.75 mm wide, 13.4 mm long, and 5.66 mm deep, overlaps a dermestid bore.

**Indeterminate Damage—**Bores on all surfaces, often conjoined and averaging 1.0–2.5 mm in diameter are from 0.75 mm deep to extend into mined cancellous tissue. A channel within a tenebrionid quarry on the medial surface about 2.0 mm wide, 5.92 mm long, and about 2.0 mm deep; a channel within a tenebrionid quarry on the medial surface about 2.7 mm wide, 4.12 mm long, and about 2.0 mm deep.

**LACMRLP 31739, *E. occidentalis*, Left Intermediate Phalanx, Pit 91**

**Dermestid Damage—**A bore extends between the poster and proximal surfaces is about 5 mm in diameter and about 9.0 mm deep; a bore extends between the anterior and proximal surfaces is about 4.5 mm in diameter, and about 7.3 mm deep; a bore extends between the anterior and posterior surfaces is about 4.0 mm in diameter and about 5.4 mm deep; a bore on the proximal surface and posterior edge is about 3.7 mm in diameter and 6.2 mm deep.

**Tenebrionid Damage—**Conjoined patches of sinuous mining between 1.5 mm, about 11.0 mm wide, and about 0.25–0.5 mm deep continue along all edges of the distal surface. A patch of sinuous mining on the distal surface is about 3.4 mm wide, 5.3 mm long, and about 0.25 mm deep; a patch of sinuous mining on the lateral surface is about 8.5 mm wide, 12 mm long, and about 0.25 mm deep.

**Indeterminate Damage—**Bores on all surfaces, often conjoined, and averaging 1.0–2.5 mm, most averaging about 1.9 mm deep, some extending up to 5.0 mm on the proximal/posterior edge. A bore on the proximal surface and the posterior edge is 1.23 mm in diameter and about 6.4 mm long. A pit on the proximal/anterior edge is 4.6 mm in diameter and 2.2 mm deep. A channel on the lateral/posterior edge is about 2.0 mm wide, about 2.0 mm deep, and at least 5.8 mm long; a channel on the posterior surface, about 1.3 mm wide, about 7.25 mm long, and about 1.0 mm deep, overlaps small bores.

**LACMRLP 50179, *E. occidentalis*, Proximal Sesamoid, Pit 91**

**Dermestid Damage—**A partial bore within the anterior side (anterior surface removed) is about 4.0 mm in diameter and at least 5.43 mm long and is overlapped by a tenebrionid quarry; a partial bore on the lateral surface about 4.0 mm wide and at least 6.88 mm deep, is overlapped by tenebrionid quarries on each side and is also conjoined by other adjacent bores; a bore on the lateral surface, about 4.5 mm in diameter and at least 2.0 mm deep, is overlapped by tenebrionid quarries on each side and is also conjoined by other adjacent bores.

**Tenebrionid Damage—**A quarry between 5.7 mm and about 23.0 mm wide, about 30 mm long, and between 3.0–15.0 mm deep encompasses all of the anterior surface except one oval 4.3 mm by 6.6 mm long and extends through to the posterior surface, as well as along the lateral, medial, and distal edges, overlaps one bore 1.89 mm in diameter and at least 2.28 mm long. A quarry on the posterior and medial edge between 2.77–7.62 mm wide, about 7.2 mm long, and between 0.25–2.18 mm deep is overlapped by another quarry; a quarry on the posterior and medial edge, about 5 mm in diameter and between 0.25–2.0 mm deep, is overlapped by another quarry; a quarry on the posterior and medial edge, between 3.6–6.14 mm wide, 9.66 mm long, and between 0.25–1.0 mm deep is overlapped by another quarry; a quarry on the posterior and lateral edge is between about 2.0–7.3 mm wide, about 21.0 mm long, and between, 0.25–6.64 mm deep.

**Indeterminate Damage—**A bore on the lateral surface 1.58 mm in diameter and at least 1.28 mm deep, is overlapped by tenebrionid quarries on each side and is also conjoined by other adjacent bores; a bore about 1.7 mm in diameter and at least 5.58 mm long within the anterior side (anterior surface removed), is overlapped by a tenebrionid quarry; two conjoined bores, both about 2.5 mm in diameter and at least 5.44 mm long within the anterior side (anterior surface removed) are overlapped by a tenebrionid quarry.

**P23-3691, *E. occidentalis*, Right Hind Proximal Phalanx, Box 1 (Figure 4B)**

**Dermestid Damage—**A channel on the posterior surface 3.45 mm wide, 13.3 mm long, and about 3.0 mm deep at each end; a medium pit on the posterior surface 3.5 mm wide and 3.0 mm deep; a medium pit on the lateral surface 3.0 mm wide and 1.9 mm deep; a medium pit on the medial surface 4.53 mm wide and approximately 3.4 mm deep.

**Tenebrionid Damage—**Surficial mining on about 50% of the proximal surface; a quarry between 2.82–7.0 mm wide, about 17.0 mm long, and between 0.5–1.38 mm deep.

**Indeterminate Damage**—A channel on the posterior surface about 2.35 mm wide and 7.17 mm long, 1.58 mm deep, intersects at a right angle with two parallel channels both about 1.5 mm wide and 3.87 mm long and about 0.5 mm deep.

**P23-5525, *Odocoileus* sp. Juvenile, Proximal Left Scapula, Box 1**

**Indeterminate Damage—**Three bores on the lateral surface about 2.5 mm in diameter and one bore approximately 1.5 mm in diameter.

**P23-7743, *E. occidentalis*, Proximal Sesamoid, Box 1**

**Dermestid Damage—**Two conjoined, medium pits on the posterior surface 3.5 mm in diameter up to 3.9 mm deep; a channel between the posterior and lateral surfaces is approximately 5.0 mm in diameter and at least 13.66 mm long (material was removed from the lateral surface).

**Tenebrionid Damage—**A quarry on the posterior surface is between about 4.08–7.0 mm wide, about 22 mm long, and between 0.25–2.85 mm deep; a quarry wraps around all surfaces is between 2.7–15.4 mm wide and about 60.0 mm long (the quarry reaches about 10.0 mm deep but overlapping traces and the great amount of mass removed make all measurements approximate).

**Indeterminate Damage—**A bore located between the articular and proximal surfaces is about 2.8 mm in diameter and had a minimum depth of 7.5 mm but surrounding material is removed. A bore on the lateral side, near the dorsal posterior surface, is about 1.2 mm in diameter and is about 3.0 mm deep but the surrounding material has been removed. A channel or partial bore about 3.6 mm wide is about 12.7 mm long but surrounding material has been removed. A channel or partial (bisected) bore about 4.5 mm wide is about 8.75 mm long but surrounding material has been removed.

**P23-7744, *E. occidentalis*, Proximal Sesamoid, Box 1 (Figure 5A)**

**Dermestid Damage—**None.

**Tenebrionid Damage—**A quarry on the posterior surface between about 7 mm–13.7 mm wide, about 28.0 mm long, and between 0.25–2.3 mm deep; a quarry between 5.5–18.4 mm wide, about 75.0 mm long, and between 0.25–10.0 mm deep wraps around the medial, proximal and lateral sides (overlapping traces and the great amount of mass removed make all measurements approximate)**.**

**Indeterminate Damage—**A bore between the proximal, posterior and anterior surfaces and about 2.5 mm in diameter with a minimum depth of 2.0 mm (the surrounding material has been removed by tenebrionids); a bore about 2.8 mm in diameter with a minimum depth of 3.68 mm next to and perhaps originally connected to the previous bore before the removal of surrounding material; five bores on the proximal, posterior, and medial sides about 1.7 mm in diameter with a minimum depth of 1.0–2.0 mm before surrounding material was removed; a channel about 2.87 mm wide with a minimum depth of 6.0 mm (surrounding material removed). A quarry on the anterior surface between about 3.5 mm and 5.63 mm wide, 15.06 mm long, and between 0.25–0.5 mm deep includes conjoined channels about 1.5 mm wide.

**P23-11272, *B. antiquus*, Left Proximal Sesamoid (Manus), Box 1 (Figure 3D)**

**Dermestid Damage—**Three small pits on the articular surface (2.24 mm by 2.8 mm, 0.8 mm deep; 3.12 mm by 3.12 mm, 0.9 mm deep; 2.9 mm by 2.9 mm; 0.5 mm deep) plus one medium pit also on the articular surface (4.3 mm by 5.3 mm; 1.5 mm deep).

**Tenebrionid Damage—**Occurs on every side and includes a quarry on the posterior surface about 9.4 mm wide, about 16.3 mm long, and from 0.25–2.3 mm deep; a quarry wraps around the perimeter of the anterior surface and extends mostly into the dorsal and lateral surface is about 3.2–9.5 mm wide and about 30.0 mm long; a quarry on the medial surface from 2.26–4.5 mm wide, between about 0.25–5.5 mm deep, and about 18 mm long; a quarry on the anterior surface between 3.84–6.02 mm wide, 12.09 mm long, and between 0.25–1.64 mm deep.

**Indeterminate Damage—**Five bores 1.6 mm, 1.64 mm, 2.36 mm, 2.4 mm, and 3.41 mm in diameter within a quarry in the posterior surface (possibly pupal chambers, bone mining or expanded foramina; the largest bore could be two conjoined bores); a bore 3.5 mm in diameter within a quarry on the lateral side which could be a pupal chamber, bone mining, or expanded foramen; an elliptical bore or quarry on the posterior surface 2.43 mm wide, 4.52 mm long and about 1.0 mm deep; a bore within a quarry on the medial side about 2.75 mm wide and about 1.6 mm deep.
